# Supplementary material for: Bone Morphogenetic Protein 2 Enhances Porcine Beige Adipogenesis via AKT/mTOR and MAPK Signaling Pathways
Source: Int J Mol Sci. 2024 Mar 31;25(7):3915. doi: 10.3390/ijms25073915 (PMC11012093; doi:10.3390/ijms25073915)
Supplement: Supplementary file 1 [file ijms-25-03915-s001.zip › supplementary information.pdf]

Supplementary Figures and Tables

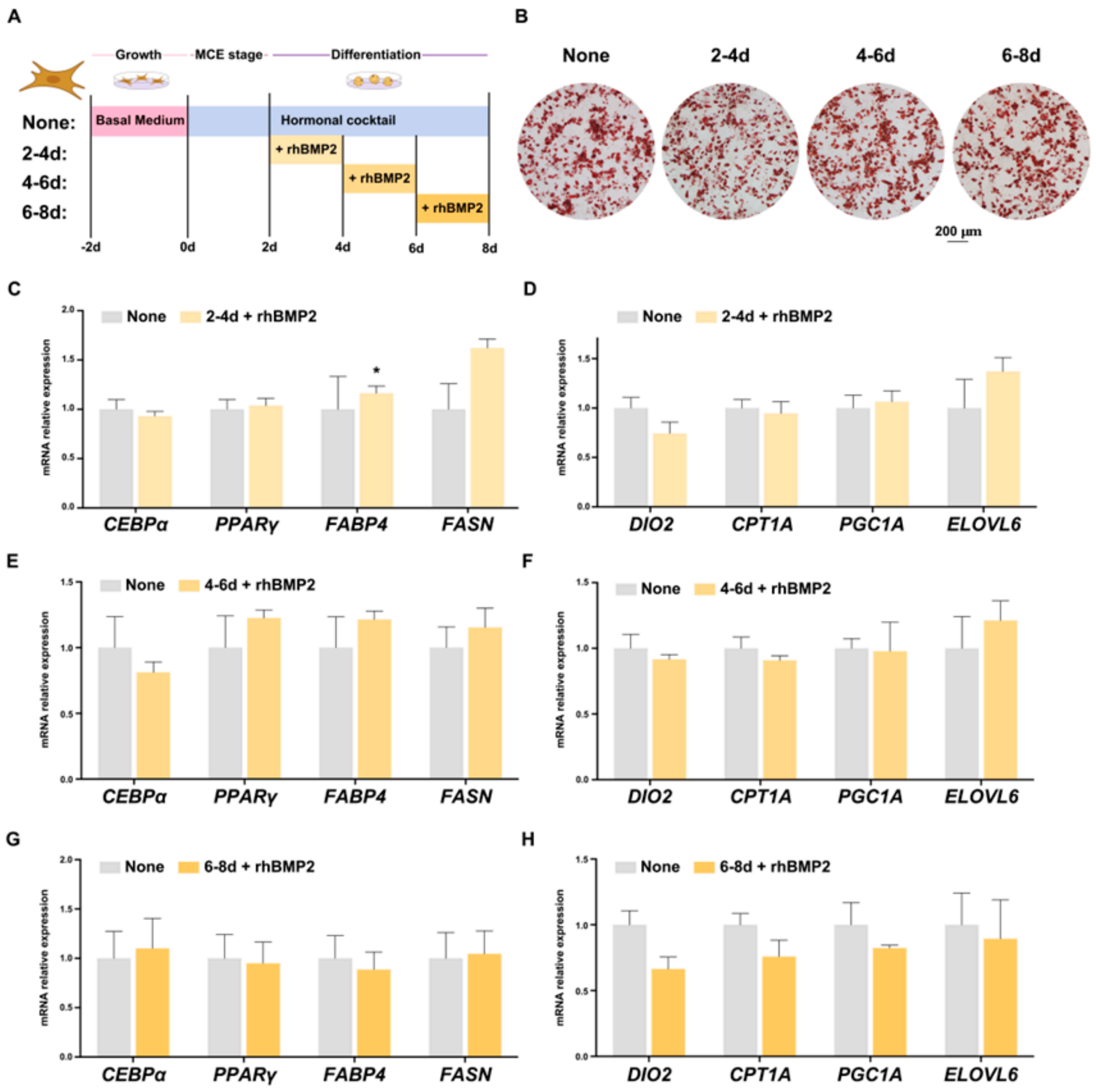

Figure S1 Pretreatment with rhBMP2 during later stages of differentiation not enhanced porcine SVFs adipogenic and thermogenic capacity

(A) Schematic representation of the experimental design. **(B)** Adipogenesis assessed by Oil red O staining at day 8 of differentiation. Adipogenesis-related genes expression by qRT-PCR at 8 days of differentiation in 2-4d **(C)**, 4-6d **(E)**, and 6-8d **(G)** treatment of rhBMP2. Beige adipocytes related genes expression by qRT-PCR at 8 days of differentiation in 2-4d **(D)**, 4-6d **(F)**, and 6-8d **(H)** treatment of rhBMP2. All values are expressed as the mean ± SEM. * $p < 0.05$, ** $p < 0.01$ and *** $p < 0.001$ were used as statistical significance standards.

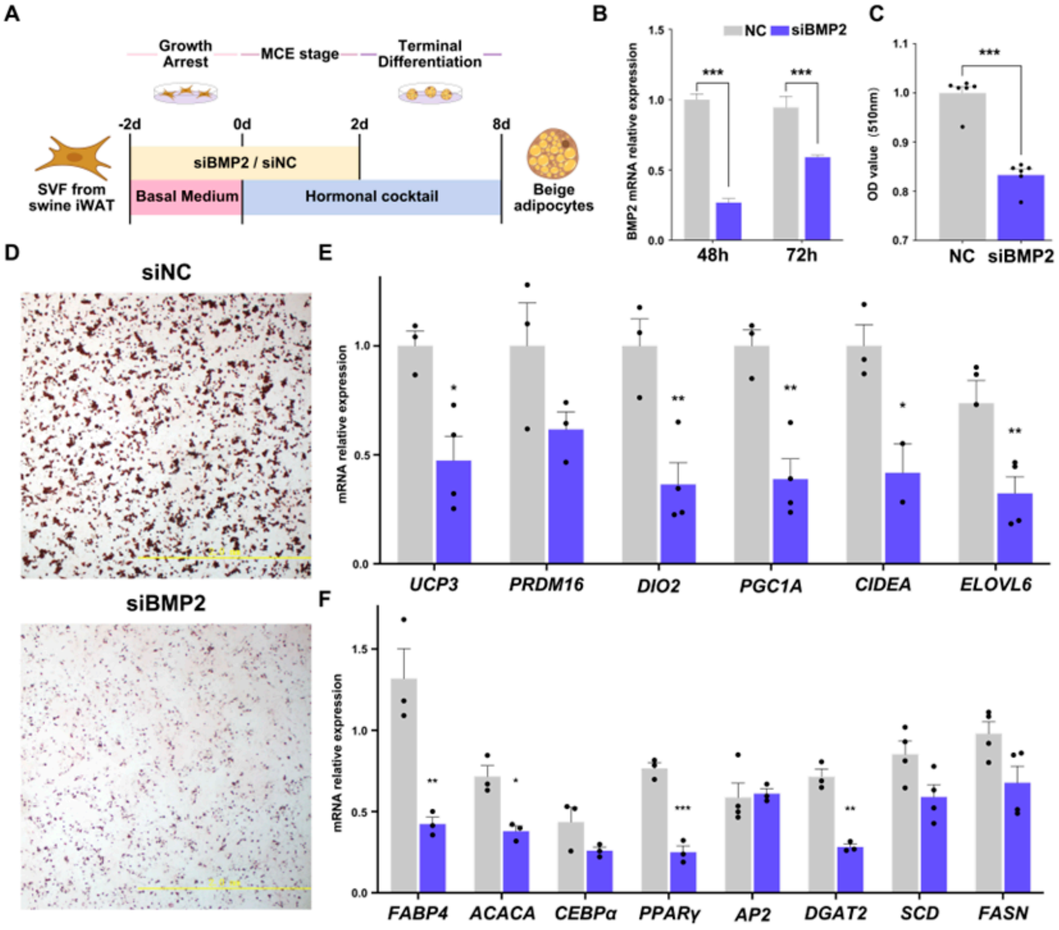

Figure S2 Knockdown *BMP2* in the early stage of differentiation reduced porcine SVFs adipogenic and thermogenic capacity

(A) Schematic representation of the experimental design. **(B)** siBMP2 efficiency in porcine SVFs. **(C)** Quantitative analysis of the Oil Red-O staining data show in D via OD measurements. **(D)** Adipogenesis assessed by Oil red O staining at day 8 of differentiation. **(E)** Thermogenesis, beige adipocytes genes, and **(F)** adipogenesis-related genes expression by qRT-PCR at 8 days of differentiation. All values are expressed as the mean ± SEM. * $p < 0.05$, ** $p < 0.01$ and *** $p < 0.001$ were used as statistical significance standards.

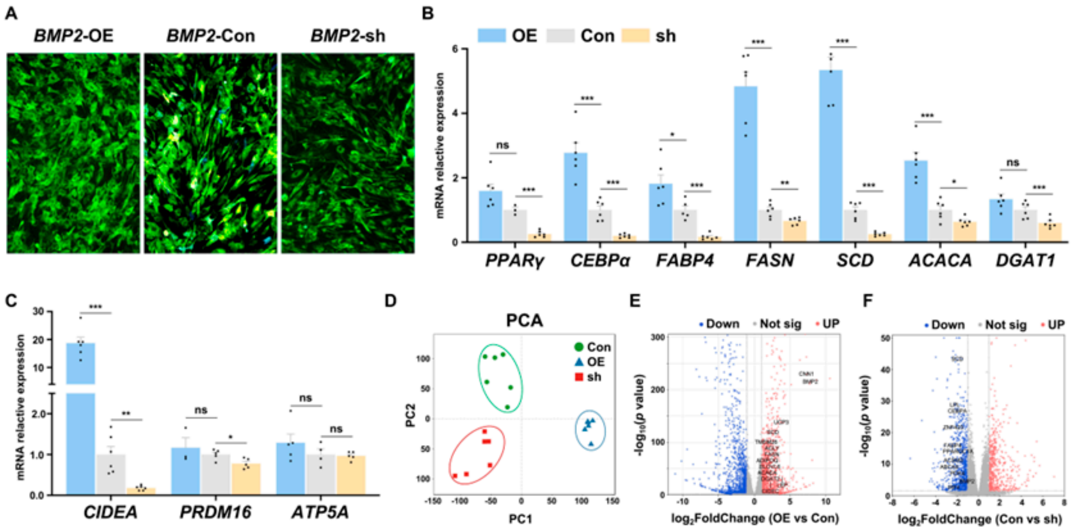

Figure S3 *BMP2* positively regulated the porcine beige adipogenic differentiation in vitro

(A) The microscopy results indicated that the transduction efficiencies of the plasmids were high and comparable among groups. **(B)** Adipogenesis-related genes and **(C)** thermogenesis-related genes expression by qRT-PCR at 8 days of differentiation. All values are expressed as the mean ± SEM. ns $p > 0.05$, * $p < 0.05$, ** $p < 0.01$, and *** $p < 0.001$ were used as statistical significance standards. **(D)** PCA of transfected with empty plasmid (Con), *BMP2* overexpressed (OE), and *BMP2* knockdown (sh) porcine SVFs (after 8 days of beige adipogenic differentiation). **(E,F)** Volcano plots showing significance on the y-axis ($-\log_{10} p$ value) plotted against the gene expression ratio ($\log_2$FC) on the x-axis; the $p < 0.05$ significance level is indicated by gray dashed horizontal lines.

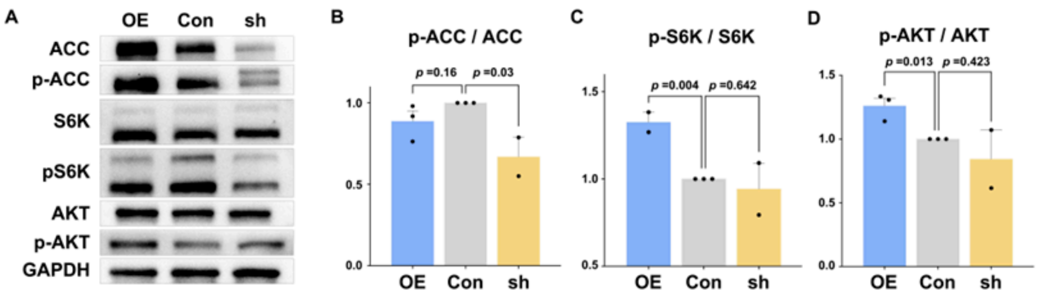

Figure S4 ***BMP2*****-overexpressed cells exhibited induced protein levels of phospho-ACC, phospho-AKT, and phospho-S6, while BMP2-deficient cells exhibited reduced protein levels**

(A) Representative Western blot showed the total protein and phosphorylation levels of ACC, S6K, and AKT in rhBMP2-treated SVFs after 8 days differentiation. Data quantification of the phosphorylation levels of ACC **(B)**, S6K **(C)**, and AKT **(D)**/ total protein. Data are presented as the mean ± SEM.

Table S2. The information of antibodies

Antibody	Dilution	Cat. #	Supplier
C/EBPβ (H-7)	1:2000	#sc-7962	Santa
FASN	1:2000	#10624-2-AP	Proteintech
DIO2	1:2000	#26513-1-AP	Proteintech
ACC	1:2000	#67373-1-Ig	Proteintech
PCNA	1:2000	#2586T	CST
PPARγ	1:4000	#2443S	CST
Phospho-ACC (ser79)	1:2000	#11818s	CST
Phospho-C/EBPβ (Thr235)	1:2000	#3084	CST
β-tubulin	1:2000	#2146S	CST
GAPDH	1:2000	#2118	CST
p44/42 MAPK (Erk1/2)	1:2000	#4695T	CST
Phospho-p44/42 MAPK (Erk1/2)	1:2000	#4370T	CST
P38 MAPK	1:2000	#8690T	CST
Phospho-p38 MAPK	1:2000	#4511T	CST
AKT	1:2000	#9272S	CST
Phospho-Akt (S473)	1:2000	#9271S	CST
P70S6K (4907)	1:2000	#2708S	CST
p70 S6kinase	1:2000	#2708S	CST
PPARGC1A	1:2000	#ab106814	Abcam
Prdm16	1:4000	#ab106410	Abcam
CEBPα	1:2000	#ab40764	Abcam
Total OXPHOS	1:5000	#ab110413	Abcam
anti-rabbit IgG HRP-conjugated antibody	1:2000	#7074S	CST
HRP-conjugated Affinipure Goat Anti-Mouse IgG(H+L)	1:2000	#SA00001-1	Proteintech

Table S3. The primer sequences of qPCR genes

Gene name	Primer sequences
PPARγ-F	CCAGCATTTCCACTCCACACTA
PPARγ-R	GACACAGGCTCCACTTTGATG
18S-F	GTAACCCGTTGAACCCCATT
18S-R	CCATCCAATCGGTAGTAGCG
CEBPα-F	GGCCAGCACACACACATTAGA
CEBPα-R	CCCCCAAAGAAGAGAACCAAG
FABP4-F	AAGAAGTGGGAGTGGGCTTT
FABP4-R	TTCCTGGCCCAATTTGAAGG
CIDEA-F	GTCAAGGCCACCATGTACGA
CIDEA-R	AGCATTCGGAGCATGTACGT
BMP2-F	GACACCCTCTGCACAAAAGG
BMP2-R	TCATTCCAGCCCACATCACT
FASN-F	CTGATCAAGGTGCTGCTGTC
FASN-R	CGAAGGAGTTTATGCCCACG
SCD-F	CTTCCTGATCATTGCCAACA
SCD-R	GCAAACCACCCTTCTCTTTG
ACACA-F	CGTGCAATCCGGTTTGTTGT
ACACA-R	TGTTGTTGTTTGGGCCTCCT
DGAT1-F	CCCACCATCCAGAACTCCAT
DGAT1-R	CGGTCTCCAAACTGCATGAG
PRDM16-F	GTGACGACCAGAACCTCACC
PRDM16-R	GACCAGCAGCTCTTCTCCTG
ELOVL6-F	CCGGAAGTTTGCCATGTTCA
ELOVL6-R	GCAGAAGAGCACAAGGTAGC
DIO2-F	CCTCTTCCTGGCGCTCTATG
DIO2-R	GTAGGCATCGAGGAGGAAGC
CD137-F	ACTGTGCTGCTGGTCATGAA
CD137-R	AACACCTTCACACTTCCTGCA
PGC1A-F	TGTGACCACTGAGAATGAGGC
PGC1A-R	TGGTTTGCATGGTTCTGGGT
AP2-F	CACTAGTGGAGGGAGAAGCC
AP2-R	CACTTGCTCATTGGGATCGG
DGAT2-F	CCCTCATAGCTGCCTACTCC
DGAT2-R	GAGGAAAGACAGGACCCACT
CPT1A-F	TTTCAGGCCGCAAAACCATG
CPT1A-R	CGCGAAATCTTGTGCCAGAG
PCNA-F	TTCCACCACCATGTTCGAGG
PCNA-R	TGAGCTGCACCAAAGAGACA
SREBP-1C-F	TTTCTGACCCGCTTCTTCCT

SREBP-1C-R	ACGGAACAACTGAGTCACCT
